# A potential pathogenic hypoxia-related gene HK2 in necrotizing enterocolitis (NEC) of newborns

**DOI:** 10.1186/s12887-022-03664-w

**Published:** 2022-10-26

**Authors:** Yujie Han, Xianghong Liu, Lili Kang, Dong Chen, Yongqing Li, Huiping Zhang, Mingying Sun, Hui Gao, Zhongtao Gai, Xiaoying Li

**Affiliations:** 1 Department of Neonatal, Children’s Hospital Affiliated to Shandong University/Jinan Children’s Hospital, No. 23976 Huaiyin District, Jinan, 250022 Shandong, People’s Republic of China; 2 Department of Neonatal, LaoLing Maternity and Child Health Care Hospital, 118 Anju Road, Laoling County, Dezhou, Shandong Province, 253600 People’s Republic of China

**Keywords:** Necrotizing enterocolitis, HK2, Hypoxia, Carbohydrate metabolism, Whole transcriptome RNA sequencing

## Abstract

**Background:**

Necrotizing enterocolitis (NEC) is a disastrous gastrointestinal disease of newborns, and the mortality rate of infants with NEC is approximately 20%-30%. The exploration of pathogenic targets of NEC will be conducive to timely diagnosis of NEC.

**Methods:**

The whole transcriptome RNA sequencing was performed on NEC samples to reveal the expression of lncRNAs, circRNAs, miRNAs and mRNAs. Using differential expression analysis, cross analysis, target prediction, enrichment analysis, the pathogenic ceRNA network and target was found.

**Results:**

Preliminarily, 281 DEmRNAs, 21 DEmiRNAs, 253 DElncRNAs and 207 DEcircRNAs were identified in NEC samples compared with controls. After target prediction and cross analyses, a key ceRNA regulatory network was built including 2 lncRNAs, 4 circRNAs, 2 miRNAs and 20 mRNAs. These 20 mRNAs were significantly enriched in many carbohydrate metabolism related pathways. After cross analysis of hypoxia-, carbohydrate metabolism-related genes, and 20 core genes, one gene HK2 was finally obtained. Dendritic cells activated were significantly differentially infiltrated and negatively correlated with HK2 expression in NEC samples.

**Conclusions:**

The promising pathogenic hypoxia-related gene HK2 has been firstly identified in NEC, which might also involve in the carbohydrate metabolism in NEC.

**Supplementary Information:**

The online version contains supplementary material available at 10.1186/s12887-022-03664-w.

## Background

Necrotizing enterocolitis (NEC) is a disastrous gastrointestinal disease of newborns, especially affecting those neonates with very-low birth weight (< 1500 g) [1]. Although the management of preterm infants has been much improved, the incidence of NEC among newborns is still worrying [2]. The beginning symptoms of NEC might be slow and insidious, such as feeding intolerance, while they can rapidly develop into a fulminant NEC [3]. The mortality rate of infants with NEC is approximately 20%-30%, and the rest survivors will face many short- or long-term complications, including poor growth, intestinal problems, and so on [4, 5]. Multiple risk factors involve in the progression of NEC, comprising prematurity, intestinal bacterial dysbiosis, formula feeds, etc. [6]. Moreover, systemic hypoxia and intestinal ischemia are main stresses leading to NEC [7]. However, potential pathogenesis of NEC complicatedly results from many factors, such as the imbalance between anti-inflammatory and proinflammatory factors [8], which still remains to be clarified. Additionally, due to nonspecific symptoms of NEC, it is hard to make early clinical diagnosis and distinguish NEC from other diseases with similar features [9]. Therefore, the exploration of promising pathogenic targets of NEC will be conducive to the timely diagnosis of NEC, indirectly contributing to better prognosis of NEC newborns.

Prematurity and enteral feeding are known as two main risk factors for NEC [10]. The primary transport function of intestinal epithelial cells is driven by consuming great amounts of energy and oxygen [11]. The digestion and nutrient transport after feeding demands more energy and oxygen. Intestinal epithelium is suffering from dynamic splanchnic circulation and anoxic environment of intestinal lumen at the same time [12], resulting in a highly fluctuating oxygen supply, thereby leading to intestinal mucosa hypoxia [11]. It has been evidenced that the synergistical role of feeding and postprandial hypoxia leads to the intestinal hypoxia in NEC [7]. Moreover, formula feeding has been reported to significantly associate with the intestinal hypoxia in NEC [13]. Compared with spontaneous intestinal perforation samples, the hypoxia related genes are significantly upregulated in human NEC samples [14]. Additionally, hypoxia and gavage feeding treatments are commonly used for NEC mouse model construction [15]. More importantly, hypoxia inducible factors (HIFs), HIF-1 and HIF-2, play crucial roles in many inflammatory bowel diseases [16]. However, the interaction of coding RNAs and non-coding RNAs has been seldom reported in hypoxia of NEC.

With the great development of next generation sequencing, whole transcriptome RNA sequencing has been contributing to reveal the crucial role of coding RNAs and non-coding RNAs in various diseases, including NEC. Herein, the purpose of our study is to explore the important pathogenic genes involving hypoxia in NEC via combining whole transcriptome RNA sequencing and further bioinformatics analyses.

## Methods

### Specimen collection

A total of 6 clinical samples were collected from Children's Hospital Affiliated to Shandong University/Jinan Children's Hospital, including 3 NEC tissue samples and 3 normal tissue samples. Our experiments were approved by ethic committee of Children's Hospital Affiliated to Shandong University/Jinan Children's Hospital, in accordance with The Helsinki Declaration. The written informed consents were obtained from the guardians of all subjects.

### Data resources

GSE46619 dataset was downloaded from Gene Expression Omnibus (GEO) database (https://www.ncbi.nlm.nih.gow/geo/). Among all samples in this dataset, NEC tissue samples and normal intestinal tissue samples were picked as the validation dataset, including 5 NEC samples and 4 normal samples.

Moreover, hypoxia gene set (200 genes) and carbohydrate metabolism gene set (286 genes) were obtained from the MSigDB database (https://www.gsea-msigdb.org/gsea/msigdb/index.jsp).

### RNA extraction and sequencing

Total RNA was extracted from the tissue samples with miRNeasy Tissue/Cells Advanced Micro Kit (Qiagen, NO.217684, Shenzhen, China), and detected on Nanodrop2000. The RNA integrity was assessed by agarose gel electrophoresis, and RIN was detected on Agilent2100. The mRNA sequencing was conducted on MGISEQ2000 platform, and the reagents used included Dynabeads® mRNA Purification Kit (for mRNA Purification from Total RNA Preps, Invitrogen, NO.61006, Shanghai, China), Library Preparation VAHTS mRNA Capture Beads (Vazyme, N401-01–02, Nanjing, China), and MGIEasy Duplex UMI Universal Library Prep Set (MGI, NO.1000006383, Shenzhen, China). Next, the raw data undergone quality control in SeqPrep software (https://github.com/jstjohn/SeqPrep) in order to obtain highly qualified sequences for subsequent analysis.

### Differentially expressed RNA analysis

The differential expression analysis was performed in limma function [17] of R language (version 4.1.0, same below). The significantly differentially expressed mRNAs (DEmRNAs), miRNAs (DEmiRNAs), lncRMAs (DElncRNAs), and circRNAs (DEcircRNAs) between NEC and normal samples were screened with |log_2_FC|> 2 and P value < 0.05.

### Target relationship prediction

The target miRNAs of DEcircRNAs were predicted using CircNA database (https://awi.cuhk.edu.cn/CircNet/php/index.php) to obtain circr-pre-miRNAs, and the structure of circRNAs, miRNA response element (MRE), and RNA-binding protein (RBP) information were obtained from Cancer-Specific CircRNA (CSCD) database (http://gb.whu.edu.cn/CSCD2/#). The miRcode database (http://www.mircode.org) was used for the targeted miRNAs prediction of DElncRNAs (lnc-pre-miRNAs). Finally, the target genes (pre-mRNAs) of circr-pre-miRNAs and lnc-pre-miRNAs were predicted in miRWalk database (http://mirwalk.umm.uni-heidelberg.de/).

### Functional enrichment analysis

The crucial genes were then subjected to the enrichment analysis in “clusterProfiler” [18] in order to obtain more functional information, including Gene ontology (GO) and Kyoto Encyclopedia of Genes and Genomes (KEGG) pathway enrichment. The GO and KEGG terms with P adjust < 0.05 (adjusted with Benjaminiand Hochberg (BH) method) were considered significantly enriched terms.

### Construction of ceRNA network

Basing on the predicted interaction pairs and differentially expressed RNAs, we have constructed a circRNA-lncRNA-miRNA-mRNA ceRNA network. The complex post-transcriptional regulation in NEC could be further exhibited through the ceRNA network.

### The impact of key gene on inflammatory responses in NEC

The expression of crucial pro-inflammatory cytokines interleukin-1β (IL-1β), interleukin-6 (IL-6), Tumour Necrosis Factor-α (TNF-α) was compared between NEC samples and normal samples.

Additionally, the relative immune cell infiltration in NEC samples was analyzed using CIBERSORT [19]. According to deconvolution algorithm, the composition of immune infiltrating cell was characterized in CIBERSORT basing on gene expression matrix and preset 547 barcode genes.

## Results

### Identification of differentially expressed circRNAs, lncRNAs, miRNAs, and mRNAs in NEC

**Fig. 1:**
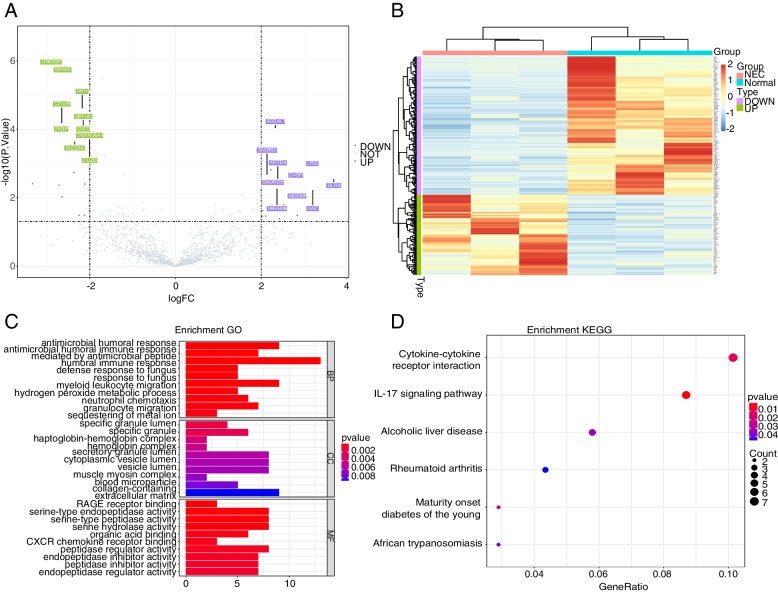
Identification of DEmRNAs between NEC vs. control samples, and their functional enrichment. **A-B** 281 significant DEmRNAs were identified. **C-D** Top 10 GO terms and all 6 significant KEGG pathways, respectively

Firstly, basing on our sequencing data, the differentially RNAs were identified between NEC and control samples. There were totally 281 DEmRNAs between NEC vs. control samples, comprising 103 upregulated DEmRNAs and 178 downregulated DEmRNAs (Fig. 1A), the expressions of which were significantly different (Fig. 1B). Considering the potential role of these 281 DEmRNAs in the onset of NEC, GO and KEGG functional enrichment was then conducted(The pathways were obtained baising on KEGG [20–22]). The 281 DEmRNAs were significantly enriched in 207 GO terms (the top 10 terms were dispalyed in Fig. 1C), and 6 KEGG pathways (Fig. 1D), such as IL-17 signaling pathway. The detailed functional results were listed in Table S1.

**Fig. 2:**
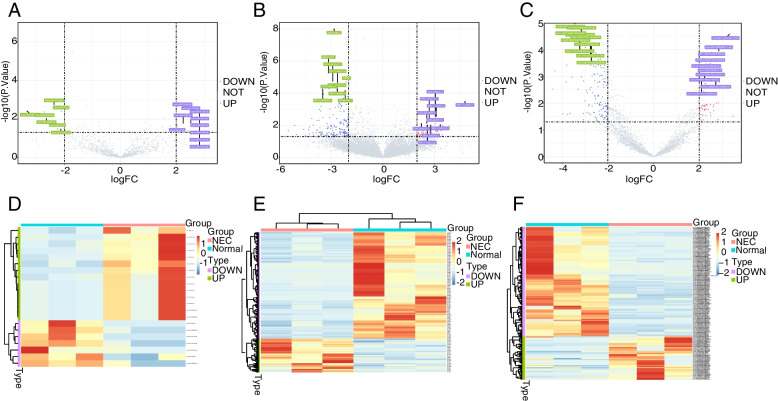
Identification of DEmiRNAs, DELncRNAs, and DEcircRNAs between NEC vs. control samples. **A-C** 21 DEmiRNAs, 253 DElncRNAs and 207 DEcircRNAs were identified, respectively. **B-D** The expressions of DEmiRNAs, DELncRNAs, and DEcircRNAs, separately

Next, differentially expressed circRNAs, lncRNAs, miRNAs were also found between NEC vs. control samples. A total of 21 DEmiRNAs, 253 DElncRNAs and 207 DEcircRNAs were found between NEC vs. control samples. Compared with control samples, 14 upregulated and 7 downregulated DEmiRNAs (Fig. 2A), 61 upregulated and 192 downregulated DElncRNAs (Fig. 2B), and 59 upregulated and 148 downregulated DEcircRNAs (Fig. 2C) were identified in NEC specimens. The expression levels of DEmiRNAs, DELncRNAs, and DEcircRNAs were significantly different between NEC vs. control samples, separately (Fig. 2D-F).

### Targeted miRNAs prediction of DElncRNAs and DEcircRNAs

**Fig. 3:**
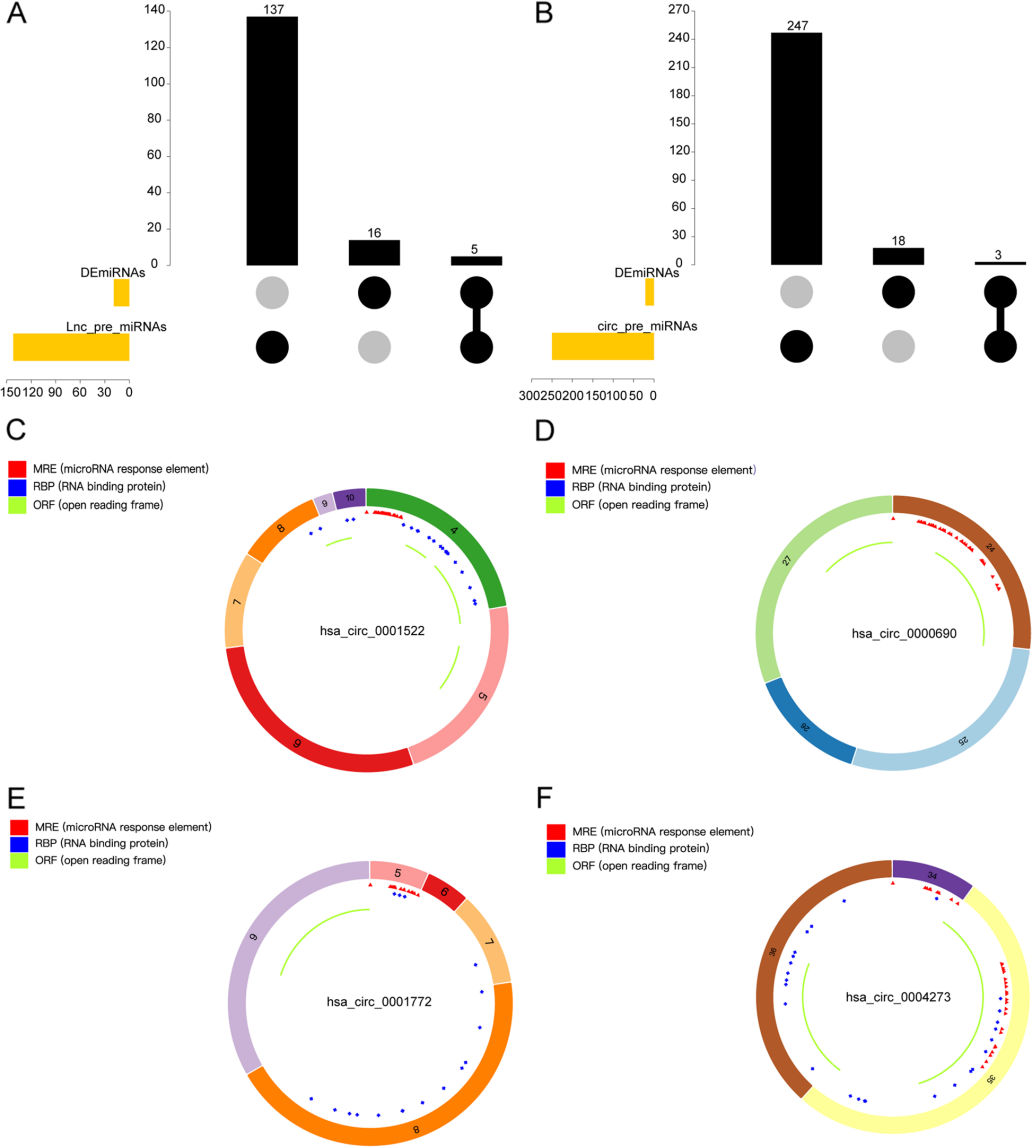
Targeted miRNAs prediction of DElncRNAs and DEcircRNAs. **A-B** Overlapped targeted miRNAs of DElncRNAs and DEcircRNAs, respectively. **C-F** The structural annotation of hsa_circ_0001522, hsa_circ_0000690, hsa_circ_0001772 and hsa_circ_0004273, separately

The targeted miRNAs of 253 DElncRNAs were predicted with miRcode database. Then regulatory pairs between 17 lncRNAs and 142 miRNAs were obtained. After cross analysis of 142 miRNAs and 21 DEmiRNAs, 5 overlapped miRNAs (lnc-pre-miRNAs) were found (Fig. 3A), including hsa-miR-124-5p, hsa-miR-222-5p, hsa-miR-518e-5p, hsa-miR-520 g-5p, hsa-miR-519-5p.

The targeted miRNAs of 207 DEcircRNAs were analyzed using circNet database and chromosomal localization. The regulatory pairs between 40 circRNAs and 250 miRNAs were identified. There were 3 overlapped miRNAs (circ-pre-miRNAs) between 250 miRNAs and 21 DEmiRNAs, including hsa-miR-222-5p, hsa-miR-522-5p, hsa-miR-520 g-5p (Fig. 3B). Basing on these 3 overlapped miRNAs, the host gene information of the corresponding 4 circRNAs was obtained, which were located on chromosomes 5, 7, 16 and 17. Moreover, the structural information and CircBaseID of these 4 circRNAs were analyzed using CSCD database, including hsa_circ_0001522 (Fig. 3C), hsa_circ_0000690 (Fig. 3D), hsa_circ_0001772 (Fig. 3E) and hsa_circ_0004273 (Fig. 3F).

### Construction of key ceRNA regulatory network

**Fig. 4:**
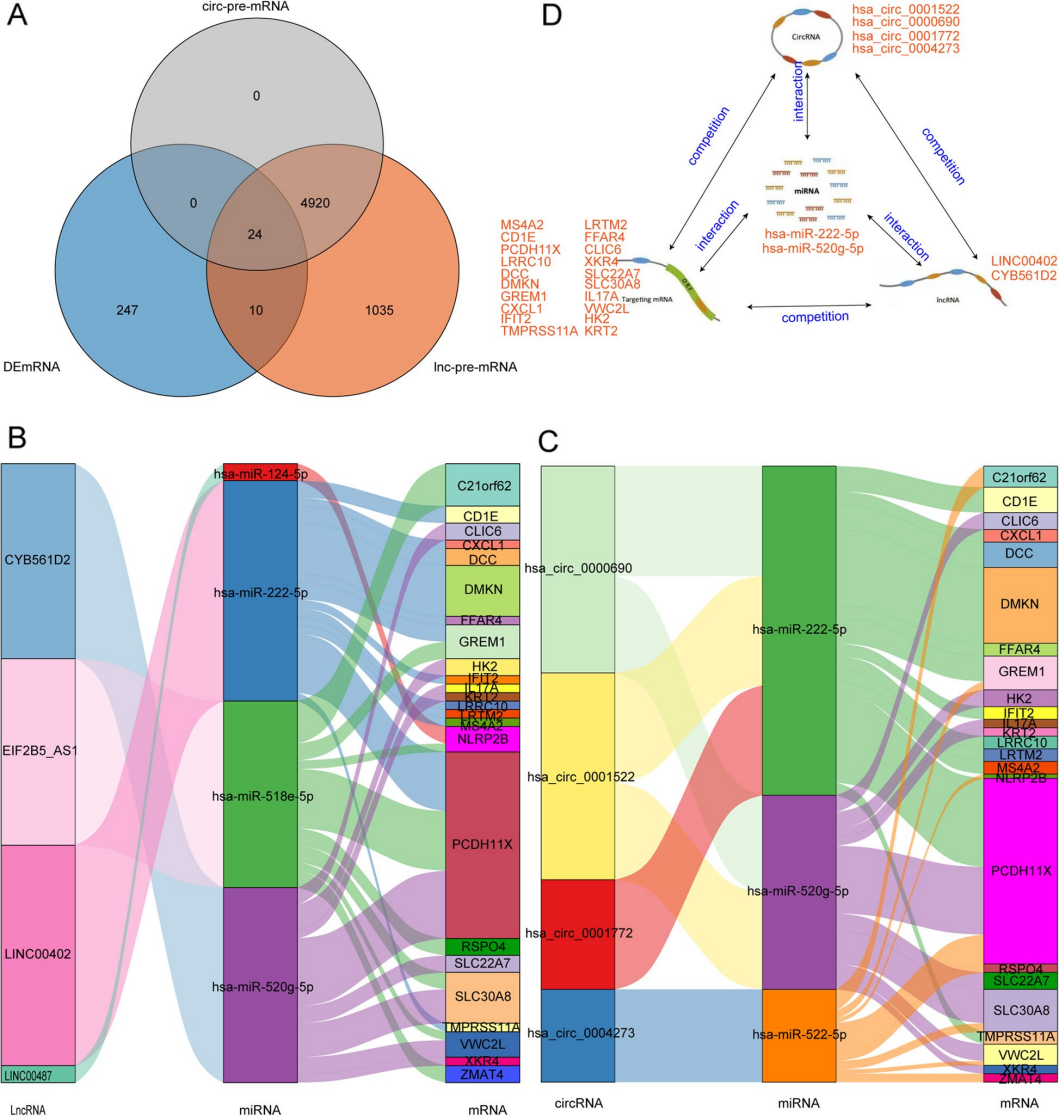
Construction of key ceRNA regulatory network. **A** The Venn diagram of pre-mRNAs and 281 DEmRNAs. **B** Correlation network of 4 lncRNAs-4 miRNAs-24 mRNAs. **C** Correlation network of 4 circRNAs-3 miRNAs-24 mRNAs’ network. **D** Key ceRNA regulatory network

Subsequently, the target genes (pre-mRNAs) of 5 lnc-pre-miRNAs and 3 circ-pre-miRNAs were identified using miRwalk database, comprising 5989 and 4944 pre-mRNAs, respectively. To further find those genes related the onset of NEC, pre-mRNAs and 281 DEmRNAs were then subjected to a cross analysis, and 24 crucial genes were identified (Fig. 4A).

Basing on these 24 crucial overlapped genes, a 4 lncRNAs-4 miRNAs-24 mRNAs’ network (Fig. 4B) and a 4 circRNAs-3 miRNAs-24 mRNAs’ network (Fig. 4C) were constructed. Among them, there were 2 overlapped miRNAs and corresponding 20 mRNAs. Thus, a key ceRNA regulatory network was built including 2 lncRNAs (LINC00402 and CYB561D2), 4 circRNAs (hsa_circ_0001522, hsa_circ_0000690, hsa_circ_0001772, and hsa_circ_0004273), 2 miRNAs (miR-222-5p and miR-520 g-5p) and 20 mRNAs (Fig. 4D), which played a crucial role in the onset of NEC.

### Functional enrichment analysis of core genes in NEC

**Fig. 5:**
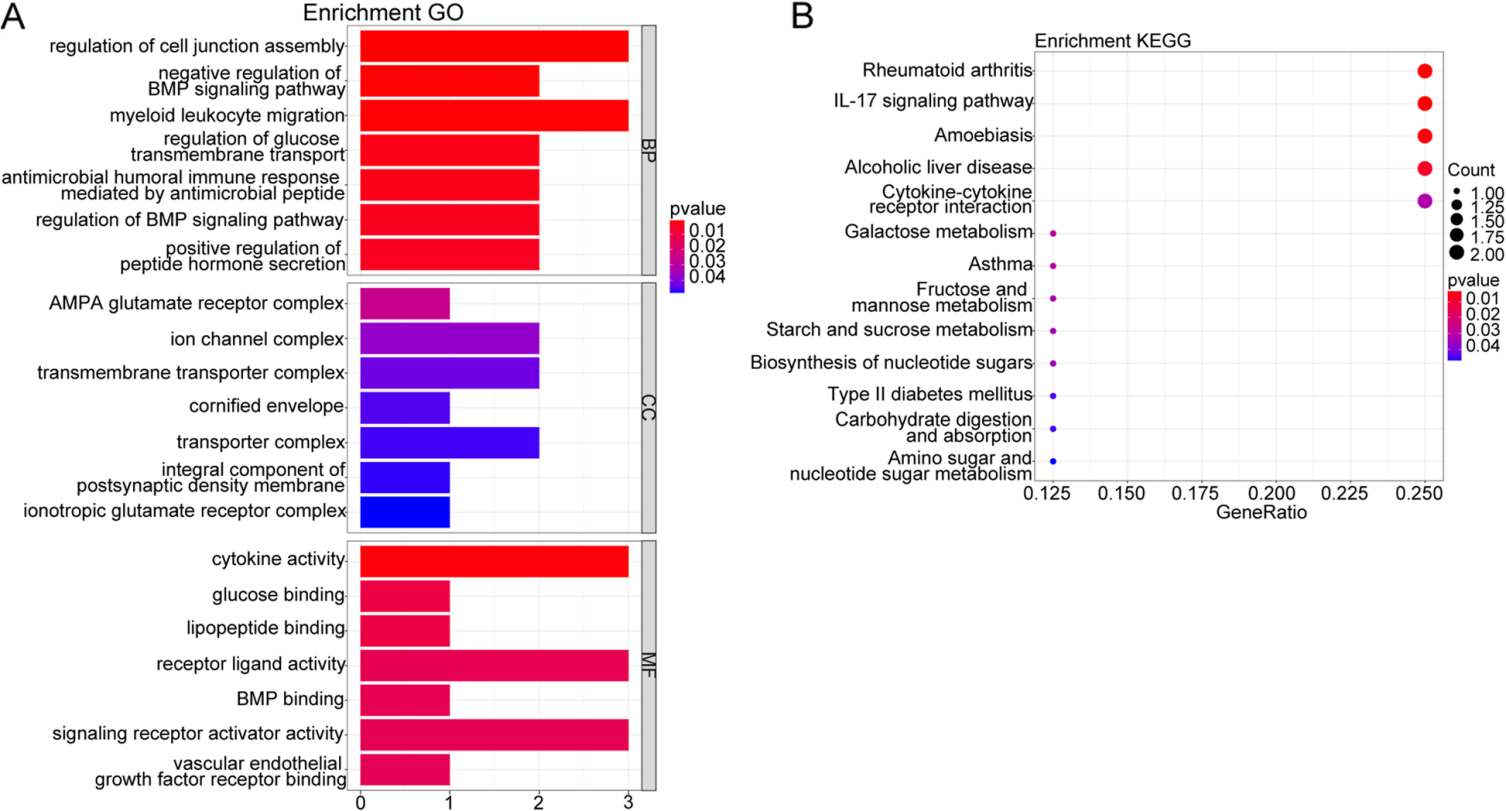
GO and KEGG functional enrichment analysis of 20 core mRNAs. **A** The top 21 significantly enriched GO terms. **B** 13 significantly enriched KEGG pathways

Due to the potentially important role of the ceRNA network in NEC, the 20 mRNAs then undergone GO and KEGG functional enrichment analysis. We found that 38 GO terms, involving Regulation of glucose transmembrane transport, Myeloid leukocyte migration, etc., were significantly (top 21 terms showed in Fig. 5A). These 20 mRNAs were significantly enriched in 13 KEGG pathways, such as Galactose metabolism, Starch and sucrose metabolism, Carbohydrate digestion and absorption, and so on (Fig. 5B). All enrichment results were shown in Table S2.

### Crucial gene HK2 involving carbohydrate metabolism and hypoxia in NEC

**Fig. 6:**
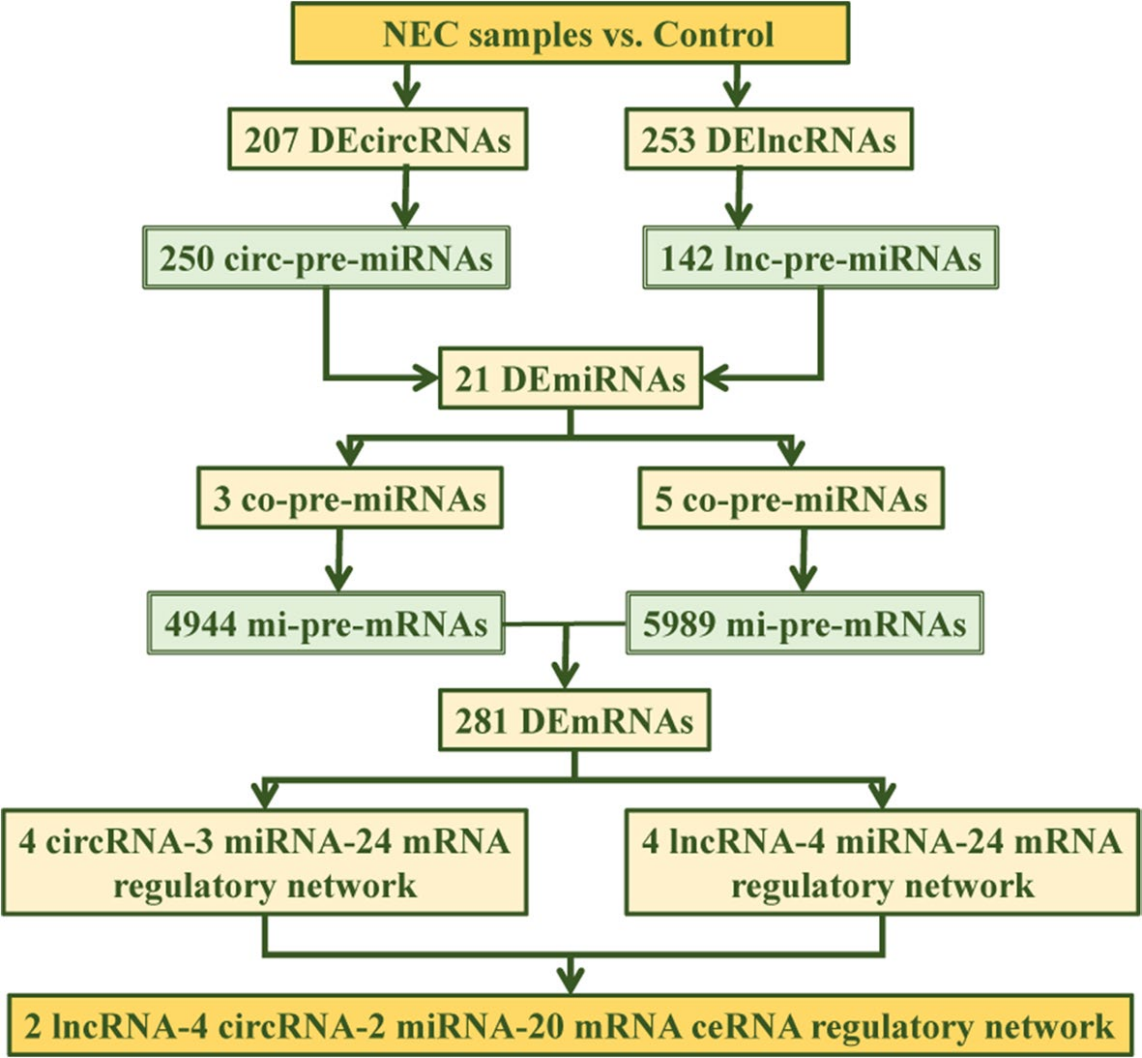
Key gene screening process

The functional enrichment results of the 20 core genes in ceRNA network implied many carbohydrate metabolism related pathways were significantly enriched, including Galactose metabolism, Fructose and mannose metabolism, Starch and sucrose metabolism, Biosynthesis of nucleotide sugars, Carbohydrate digestion and absorption, and Amino sugar and nucleotide sugar metabolism. Moreover, undigested carbohydrate fermentation in bowel has been demonstrated to yield short chain organic acids, thereby leading to intestinal mucosa disruption and inflammation [23]. The functional information inspired us regarding the core genes’ impacts on carbohydrate metabolism in NEC. On the other hand, combining the crucial role of hypoxia in NEC [13], the crucial genes involving carbohydrate metabolism and hypoxia in NEC were screened, and the whole screening process was shown in Fig. 6.

**Fig. 7:**
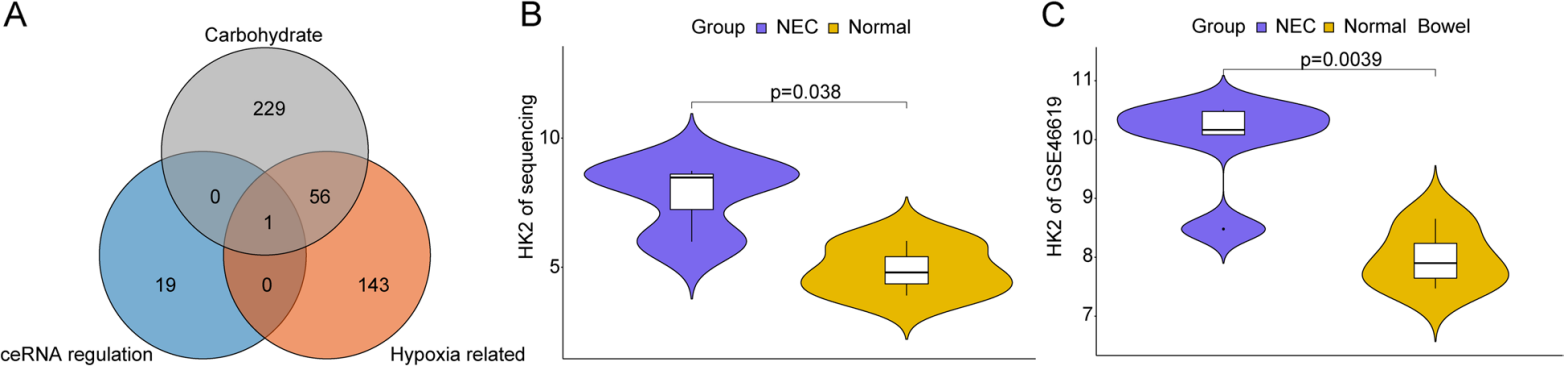
Screening of crucial gene HK2 involving carbohydrate metabolism and hypoxia in NEC. **A** Results of cross analysis. **B-C** The expression of HK2 in NEC samples in local sequencing data and GSE46619, respectively. *P* value was calculated with *t*-test

Then hypoxia related gene set (200 genes) and carbohydrate metabolism gene set (286 genes) were downloaded from MSigDB database (Table S3). After cross analysis of hypoxia related gene set, carbohydrate metabolism gene set, and 20 core genes, one gene HK2 was finally obtained (Fig. 7A).

In our sequencing data, HK2 was significantly highly expressed in NEC tissues comparing with controls (Fig. 7B). The expression of HK2 was also evaluated in an independent cohort GSE46619, and significantly higher HK2 expression was also observed in NEC samples (*p* < 0.01) (Fig. 7C). Collectivelly, HK2 was a probably crucial pathogenic gene in NEC involving carbohydrate metabolism and hypoxia.

### Impact of HK2 on inflammatory responses in NEC

**Fig. 8:**
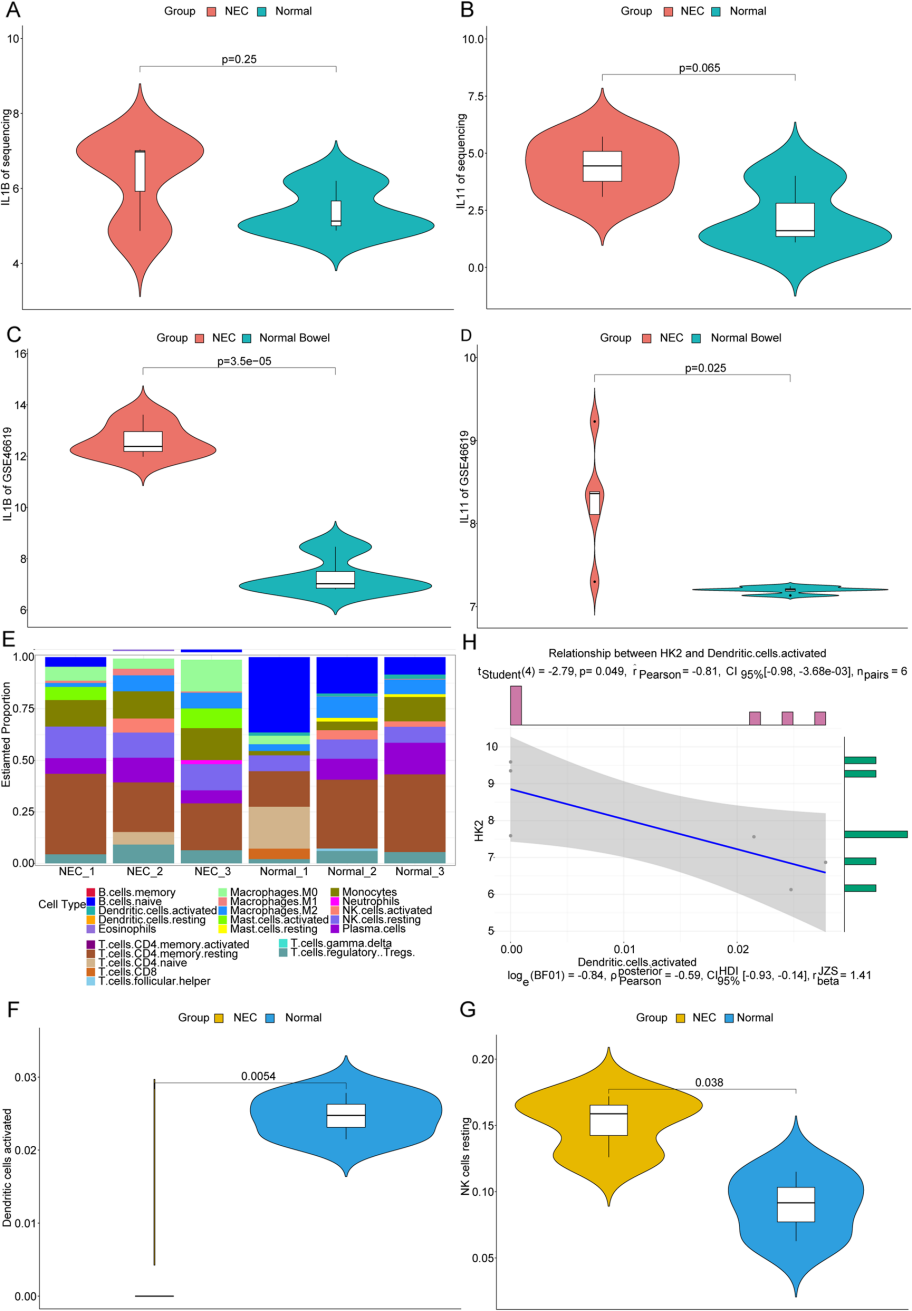
Potential impacts of HK2 on inflammatory responses in NEC. **A-B** In our sequencing data, the expression levels of IL-1β and IL11, respectively. **C-D** In dataset GSE46619, the expression levels of IL-1β and IL11, respectively. **E** The immune cell infiltration in our local samples. **F-G** Dendritic cells activated and NK cells resting were significantly differentially infiltrated between NEC vs. controls, separately. **H** The correlation analysis of HK2 and Dendritic cells activated. *P* value was calculated with *t*-test

The enhancement of immune and inflammation responses are important hallmarks of NEC [24]. Thus, the potential impacts of HK2 on inflammatory responses in NEC were also analyzed herein. Firstly, crucial proinflammatory cytokines’ expression was compared between NEC vs. Controls using our sequencing data. Our results indicated that the expression levels of IL-1β and IL11 showed higher expressions in NEC samples compared with normal samples (Fig. 8A-B). Moreover, the same tendency was also found in GSE46619 dataset (Fig. 8C-D). Our data suggested that inflammatory responses were probably activated in NEC samples.

Additionally, basing on our sequencing data, the immune cell infiltration in all samples was also evaluated in CIBERSORT to get more information regarding immune responses. The general results of 22 types of immune cells’ infiltration in NEC and control samples were shown in Fig. 8E, indicating the individual characteristics. Additionally, we found that two types of immune cells, NK cells resting and Dendritic cells activated were significantly differentially infiltrated between NEC vs. Controls (Fig. 8F-G), which probably contributed to the development of NEC. Besides, in NEC specimens, there was significant negative correlation between HK2 and infiltration of Dendritic cells activated (Fig. 8H).

## Discussion

As a leading cause of neonatal mortality, NEC has been a great healthy threat to neonates, especially to premature infants [25]. However, to date, the comprehensive analysis of mRNAs, miRNAs, lncRMAs, and circRNAs in NEC tissue specimens of newborns is quite limited. Accordingly, we herein utilized the whole transcriptome RNA sequencing and further bioinformatics mining to study the potential pathogenic ceRNA network and crucial gene in NEC. HK2 was found to involve in hypoxia and carbohydrate metabolism in NEC.

Although the role of some non-coding RNAs has been explored in NEC rat models, for example, miR-27a-5p and miR-187-3p are probably involving the NEC pathophysiological mediation through Wnt signaling [6]. However, little is known about the role of lncRMAs, circRNAs, and their interaction with mRNAs. Accordingly, whole transcriptome RNA sequencing was conducted to revealed the potential pathogenic non-coding RNAs and mRNAs in NEC. Preliminarily, 281 DEmRNAs, 21 DEmiRNAs, 253 DElncRNAs and 207 DEcircRNAs were identified in NEC samples. The enrichment analysis indicated that the 281 DEmRNAs were significantly enriched in IL-17 signaling pathway. IL-17 signaling pathway is able to trigger several pro-inflammatory molecule events, such as IL22, IL1β, TNF, and so on [26]. The main pathophysiology of NEC has been known as extreme inflammatory responses and necrosis [27]. Although IL-17 signaling has been illustrated in some IBDs like Crohn’s disease [26], it has been seldom studied in NEC. Our data provided more evidence regarding inflammatory responses in NEC. Furthermore, we found that proinflammatory cytokines, IL-1β and IL11, showed higher expression in NEC. Besides, Dendritic cells (DCs) activated were significantly differentially infiltrated between NEC vs. Controls, and negatively correlated with the HK2 expression in NEC samples. Emami et al. have reported that in NEC mouse models, C. sakazakii infection could destruct the intestinal epithelium via recruiting more DCs and suppressing the DC maturation [28]. Obviously, lower levels of activated DCs seemed to exert unfavorable effects on NEC.

Subsequently, after target prediction and cross analyses basing on the differentially expressed RNAs, a key ceRNA regulatory network was built including 2 lncRNAs (LINC00402 and CYB561D2), 4 circRNAs, 2 miRNAs (miR-222-5p and miR-520 g-5p) and 20 mRNAs. Most of the non-coding RNAs are reported in NEC for the first time in our study. Further functional information of these 20 key genes was obtained, involving many carbohydrate metabolism related pathways. Additionally, considering the important effects of hypoxia on NEC of newborns, the core gene HK2 was finally screened involving carbohydrate metabolism and hypoxia in NEC. Although limited reports directly supported the interaction between HK2 and these non-coding RNAs, especially in NEC, some indirect clues could be found. LncRNA CYB561D2 has been indicated to activate STAT3 and lead to the immunosuppression in brain tumor [29]. Meanwhile, hypoxia was a crucial factor driving immunosuppression in various tumors [30]. However, whether CYB561D2 and HK2 could exert similar roles under hypoxia in NEC should be further investigated. Recently, in a breast cancer cell line, miR-222-5p has been evidenced to be downregulated under hypoxia [31], indicating that miR-222-5p expression indeed was affected by hypoxia. Whereas, the interaction between HK2 and miR-222-5p under hypoxia still needs to be further clarified in NEC in the future.

Hexokinases (HKs) catalyze glucose to yield glucose-6-phosphate (G6P) involving the first committed step in glucose metabolism, and HK2 (hexokinase 2) is a member of hexokinase family [32]. Although HK2 has been seldom studied in NEC of newborns, the role of HK2 has been illustrated in many diseases. Han et al. have recently demonstrated that differential DNA methylation/ hydroxymethylation and gene expression of HK2 was observed in mouse model of inflammatory bowel disease (IBD) [33]. Whereas, as another intestinal inflammatory disease, whether similar epigenetic modification of HK2 occurs in NEC still needs to be clarified. Additionally, in hepatomas, HK2 has been found to be highly expressed in tumor cells, accompanied with great G6P production, and G6P was an important carbon and energy source in hypoxic conditions [34]. More importantly, as a crucial transcription factor in hypoxia, HIF-1α is able to bind with the promoter of HK2 to promote the transcription of HK2 [35]. Thus, we suspect that in NEC, HK2 exerts adaptive role in providing carbon and energy source in intestinal epithelium in a dynamic oxygen conditions, involving hypoxia and carbohydrate metabolism. Additionally, Pavo et al. have recently reported their findings in repetitive ischemia/ reperfusion model that HK2 was significantly upregulated in ischemic zone while downregulated in heart regions, indicating the intrinsic remote ischemic conditioning (RIC) of myocardium [36]. In NEC, RIC has been increasingly considered a promising tool to protect distant organs from ischemia/ hypoxia-induced damage [37]. Accordingly, their work inspired us to connect HK2 with RIC, which deserved subsequent investigation in the near future.

Finally, it is still urgently needed to reveal the HK2 related underlying mechanisms in NEC, in order to better understand the detailed pathogenic role of HK2. Only then it will be possible to further explore its role in larger sample size and other sample type, such as blood sample. All above work are expected to help us to apply our findings to clinical cases earlier. The hypoxia-related gene HK2 we identified is promising to provide more alternatives for early detection and diagnosis of NEC.

## Conclusions

In summary, basing on the whole transcriptome RNA sequencing analysis of our local clinical samples, we fully use bioinformatics tools and mine the potential pathogenic ceRNA network in NEC. Notably, a promising pathogenic hypoxia-related gene HK2 has been firstly identified in NEC, involving the carbohydrate metabolism. Our findings give more insights into understanding the pathogenesis of NEC of newborns.

## Supplementary Information

**Additional file 1.** Detailed functional results of DEmRNAs.

**Additional file 2.** Detailed GO and KEGG functional results of core genes in NEC.

**Additional file 3.** All genes in hypoxia gene set and carbohydrate metabolism gene set.

## Data Availability

The datasets generated and analysed during the current study are available from https://www.ncbi.nlm.nih.gov/bioproject/PRJNA835160.

## Abbreviations

NEC: Necrotizing enterocolitis
HIFs: Hypoxia inducible factors
GEO: Gene Expression Omnibus
MRE: MiRNA response element
RBP: RNA-binding protein
CSCD: Cancer-Specific CircRNA
GO: Gene ontology
KEGG: Kyoto Encyclopedia of Genes and Genomes
IL-1β: Interleukin-1β
IL-6: Interleukin-6
TNF-α: Tumour Necrosis Factor-α
